# A new xyelotomid (Hymenoptera) from the Middle Jurassic of China displaying enigmatic venational asymmetry

**DOI:** 10.1186/s12862-016-0730-0

**Published:** 2016-08-02

**Authors:** Taiping Gao, Chungkun Shih, Michael S. Engel, Dong Ren

**Affiliations:** 1 College of Life Sciences, Capital Normal University, Beijing, 100048 China; 2 Department of Paleobiology, National Museum of Natural History, Smithsonian Institution, Washington, DC 20013-7012 USA; 3 Division of Entomology, Natural History Museum, University of Kansas, Lawrence, 66045 KS USA; 4 Department of Ecology & Evolutionary Biology, University of Kansas, Lawrence, KS 66045 USA

**Keywords:** Insect, Fossil, Symphyta, *Aethotoma*, Jiulongshan formation, Daohugou

## Abstract

**Background:**

Pterygota insects typically have symmetric veins in left and right wings. For studying taxonomy and phylogeny of fossil insects, venational patterns are commonly used as diagnostic characters, in conjunction with preserved body characters. Some examples of asymmetrical venation are known among extant insects, but only a few fossil insects with asymmetric wings have been reported, among which a previously described xyelotomid of Hymenoptera, *Xyelocerus diaphanous*, displays an unusual, small cell of vein Rs in the left forewing, but not in the right.

**Results:**

Herein we report a new sawfly of the family Xyelotomidae, *Aethotoma aninomorpha* gen. et sp. nov., from the late Middle Jurassic of China having a simple Sc in the forewing and Sc with two branches in the hind wing. In additional, the new specimen exhibits an enigmatic venational asymmetry. In the right forewing, crossvein 2r-rs of forms a loop, then forks into 2 long branches reaching Rs, while 2r-rs of the left forewing forks into 2 short branches reaching Rs, in contrast to a linear 2r-rs in typical fossil and extant sawflies.

**Conclusion:**

Such rare asymmetrical venation found from fossil sawflies provides a glance at early occurrences of venational variability and instability, or possibly aberrational development, for insects in the late Middle Jurassic.

## Background

In studies of morphology, taxonomy, and phylogeny within insects, individual characters are assumed to be stable and heritable, and these are in turn adopted as diagnostic traits and/or suitable for cladistics analysis [1]. However, aberrations in the developmental process have occurred since the beginning of life and are frequent in the extant groups of insects [2], which many result in altered physiology, behavior, or morphology, but are not heritable. To a certain extent such character instability causes confusion to our classification work of insects, especially for fossil insects with limited and insufficient characters as preserved. Hymenoptera, like other winged insects, exhibit bilateral symmetry, while in some cases, minute and insignificant differences can be found between the left and right wings. In the fossil records, only a few specimens of Hymenoptera have been documented to have unusual venational pattern. Lara et al. recently reported a xyelid sawfly, *Potrerilloxyela menendezi* Lara, Rasnitysn & Zavattieri, 2014, from the Late Triassic, having a crossvein 2m-cu with a loop connecting with vein 4-M [3]. In 2008, Rasnitsyn described a Jurassic sawfly, *Shartexyela mongolica* Rasnitsyn, 2008, having a vein 2m-cu with a short stub at mid-length [4]. However, both *P. menendezi* and *S. mongolica* have only a single wing preserved and while these venational elements differ from what is expected in a sawfly forewing, the degree to whether or not these represent asymmetry or some other divergence from the standard pattern is uncertain. Gao et al. (2013) reported a huge sawfly, *Hoplitolyda duolunica* Gao, Shih, Rasnitsyn & Ren, 2013, from the Early Cretaceous of China, having a uncommon forewing that a protrudent short stub originates from vein M + Cu, but does not connect with other veins, but the similar characters are found in both of the left and right forewing [5]. Gao et al. described a xyelotomid, *Xyelocerus diaphanous*, that displays an unusual, small cell of vein Rs in the left forewing, but not in the right [6].

Herein we document an enigmatic asymmetrical venation in a late Middle Jurassic sawfly. *Aethotoma aninomorpha* gen. et sp. nov., as a member of the putatively paraphyletic family Xyelotomidae, from the Jiulongshan Formation, of Daohugou, Ningcheng County, Inner Mongolia, China.

## Methods

The specimen is housed in the Key Laboratory of Insect Evolution & Environmental Changes, College of Life Sciences, Capital Normal University, Beijing, China. Fossils were examined using a Leica MZ 16.5 dissecting microscope. Line drawings were prepared with CorelDraw X6 and Adobe Photoshop CS6. The photographs and magnified images of details of the specimens were taken with a digital camera system attached to a Leica MZ16.5. Specimens were at times treated with ethanol (95 %) on the surface to create greater contrast between the fossil and the surrounding matrix. Wing venation nomenclature used herein is based on the interpretation of Huber and Sharkey [7]. Venation abbreviations used in the text and figures: C, Costal; Sc, Subcostal; R, Radial; Cu, Cubital; A, Anal; Rs, Radial sector; M, Medial.

## Results

### Systematic palaeontology

Class Insecta Linnaeus, 1758

Order Hymenoptera Linnaeus, 1758

Superfamily Tenthredinoidea Latreille, 1802

Family Xyelotomidae Rasnitsyn, 1968

*Aethotoma* gen. nov.

*Type and only species. Aethotoma aninomorpha* sp. nov.

*Etymology*. The generic name is a combination of the Greek prefix “*aeth*-,” meaning unusual and unique, and “-*toma*,” a suffix from *Xyelotoma* Rasnitsyn, 1968. The gender of the name is feminine.

*Diagnosis*. Antenna 10-segmented, enlarged first flagellomere about 4 times as long as remaining flagellomeres combined, much thicker than remainder of flagellum. Tarsomere V longer than combined length of tarsomeres III and IV. Forewing with Sc simple, terminating into R, lacking a separate crossvein-like distal part; pterostigma fully sclerotized; first abscissa of Rs (Rs1) about 1/5 of that of M (M1). Hind wing Sc with two branches.

*Remarks*. The new genus is placed in Xyelotomidae based on the antenna with the 3rd segment very long and thick but much fewer flagellomeres. It differs from other genera of the family by the simple Sc in the forewing and Sc with two branches in the hind wing. Xyelotomidae are likely a paraphyletic assemblage and not a natural group, representing a potential stem-group to Tenthredinoidea [8]. Should this be conclusively established in future studies, then Xyelotomidae will require division into multiple groups. In additional, even the monophyletic clade of Tenthredinoidea is challenged recently, and further research is needed [9].

*Aethotoma aninomorpha* sp. nov.

(Figs. 1 and 2)

**Fig. 1:**
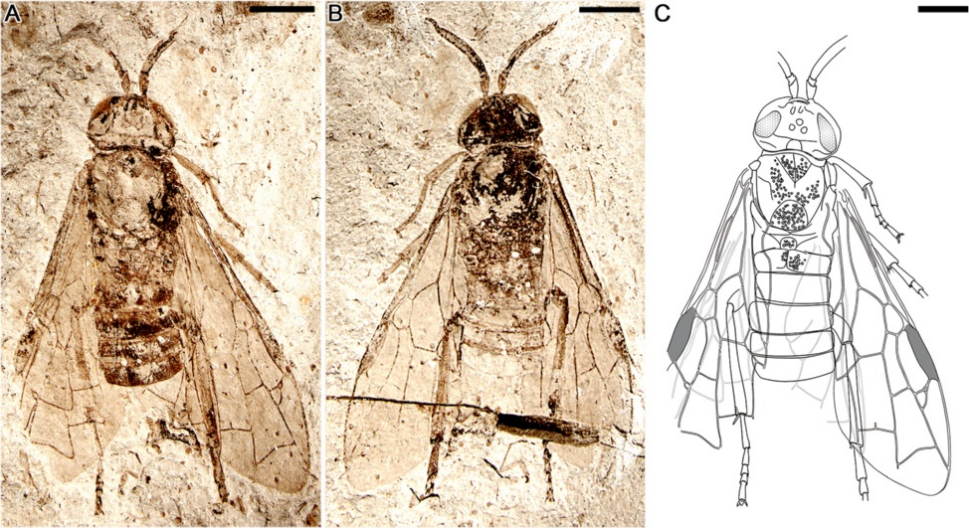
Holotype of *Aethotoma aninomorpha* gen. et sp. nov. from the Middle Jurassic (Jiulongshan Formation) of northeastern China. No. CNU-HYM-NN2012003p/c. **a** and **b**, Photographs of part and counterpart. **c**, Line drawing of part. Scale bars: 2 mm

**Fig. 2:**
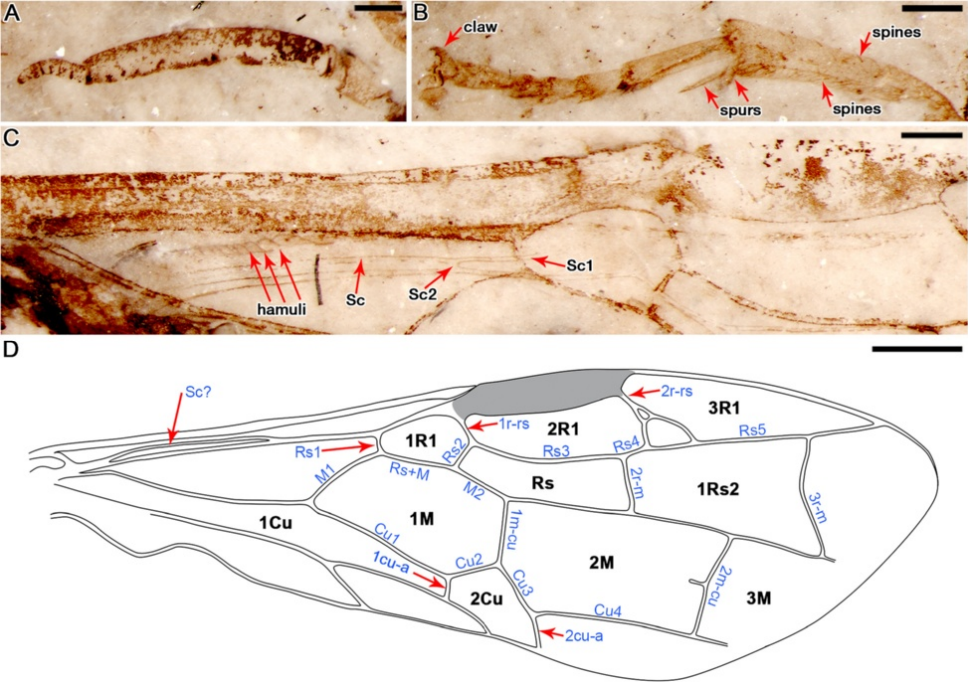
Detailed structures of holotype of *Aethotoma aninomorpha* gen. et sp. nov. No. CNU-HYM-NN2012003p/c. **a**, left antenna of the counterpart. **b**, left fore leg of the counterpart. **c**, right hind wing of the counterpart showing row of hamuli. **d**, line drawing of the right forewing of the part. Scale bar represents 0.4 mm (**a**–**c**), 1 mm (**d**)

urn:lsid:zoobank.org:act:BC73BF5C-3922-4C56-8A9D-0B3AB7028090

*Etymology*. “*Aninomorphus*” means “different shape”, referring to the unique venation of the forewings.

*Holotype*. CNU-HYM-NN2012003p/c; the fossil is complete except for the apical segments of the abdomen (the 6th segment onward); sex unknown.

*Type locality and horizon*. Collected from the Jiulongshan Formation of Daohugou Village, Shantou Township, Ningcheng County, Inner Mongolia, China, the late Middle Jurassic (Bathonian-Callovian boundary, 165 million years ago [Mya]) [10, 11].

*Description*. Body length 10.2 mm as preserved (Fig. 1). Head nearly trapezoidal, 2.8 mm wide, 1.8 mm high, compound eyes (1.2 mm high) relatively small, oval. Three ocelli present, round, together forming an equilateral triangle, closely spaced. Antenna (3.5 mm long) (Fig. 2a) much longer than width of head, scape (0.5 mm long) slightly thicker than pedicel (0.2 mm in length), twice as long as latter; first flagellomere (2.1 mm long) about 4 times as long as remaining 7 flagellomeres combined (0.6 mm), remaining flagellomeres somewhat equal in length and width, each about 1/3 width of first flagellomere.

**Fig 3:**
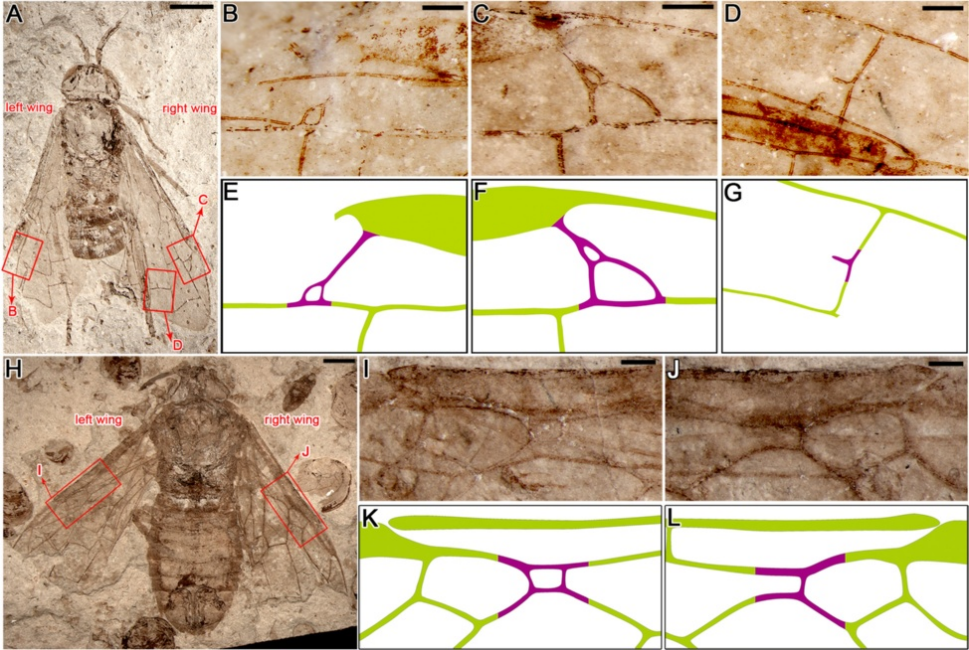
*Aethotoma aninomorpha* gen. et sp. nov. and *Xyelocerus diaphanus* display fluctuating asymmetry in the forewings, purple color highlights asymmetrical veins. **a**. Holotype of *A. aninomorpha*. **b** and **e**, vein 2r-rs of the left forewing. **c** and **f**, vein 2r-rs of the right forewing of *A. aninomorpha*. **d** and **g**, 2m-cu of the right forewing of *A. aninomorpha*. **h**, Holotype of *Xyelocerus diaphanus* from the Middle Jurassic (Jiulongshan Formation) of northeastern China. No. CNU-HYM-NN-2008011p. **i** and **k**, part of the left forewing of *X. diaphanus*. **j** and **l**, part of right forewing of *X. diaphanus*, showing normal venation. Scale bar represents 2 mm (**a** and **h**), 0.4 mm (**b**–**d**), 0.2 mm (**i** and **j**)

Forewing (Fig. 2d) Sc simple (without branches), merging into R much prior to separation of Rs from R, probably shorter than half of C in length; veins C and R markedly thickening before pterostigma (length 1.8 mm, widest part 0.4 mm), latter fully sclerotized. First abscissa of Rs (Rs1, 0.2 mm) about 1/5 length of M (M1, 1.0 mm). M + Cu slightly bent, Rs + M (0.9 mm long) nearly three times as long as 1r-rs (0.3 mm long), latter equal to section of Rs nearest it (Rs2, 0.3 mm long); 2nd abscissa of M (M2, 0.6 mm) slightly shorter than 1m-cu (0.7 mm); 2r-rs of left forewing forking into 2 short braches reaching Rs (Fig. 3a, b and e), right forewing with 2r-rs forming a small loop, then forking into 2 long braches reaching Rs (Fig. 3a, c and f). Crossvein 3r-m distinctly bent at middle, about twice as long as 2r-m. In the right forewing, a free vein (about 0.2 mm) extending from middle point of 2m-cu (Fig. 3a, d and g). Hind wing partially preserved (Fig. 2c), with Sc two-branched, Sc2 short; at least 12 basal hamuli visible at about 1/6 of hind wing length near basal part of vein C, traces of distal and intermediate group hamuli visible (but details not clear).

Thorax (3.1 mm wide at the widest point, about 4.1 mm long) slightly wider than head; where observed, thoracic dorsum with rough sculpture (densely areolate or tuberculate); pronotum (2.2 mm wide) short; mesoscutum large, anterior edge arched. Protibia with two spur present, one long and one short (Fig. 2b) (0.4 mm long); protibia 1.4 mm long, probasitarsus equal to protarsomeres II and III combined, protarsomere V longer than combined lengths of protarsomeres III and IV, pretarsus with strong claw (0.2 mm long), with sharp tooth extending from inner side of each pretarsal claw; all legs covered by numerous fine setae, each tarsus with some stiff setae apically; lengths of individual protarsomeres (mm): 0.76, 0.27, 0.20, 0.17, 0.38; metatarsomeres: 1.01, 0.53, 0.35, 0.24, 0.49; metapretarsal claws relatively short, without teeth.

## Discussion

### Evolutionary changes in venational patterns and antennal shapes and their significance in sawfly

Hymenoptera have a comparatively simple pattern of wing venation among many pterygote lineages, and it has been simplified further across the order [7]. From the putatively most basal sawflies, such as *Gigantoxyela quadrifurcata* Rasnitsyn, 1966 in the family Xyelidae [12], to the more derived, the venation has been gradually reduced, often as a result of dramatic reduction in overall body size and associated with functional changes in flight dynamics, tied to wing shape and folding [13, 14]. Xyelotomidae, a small and extinct family of sawflies, first erected by Rasnitsyn in 1968 [15], so far comprise only 21 species in 14 genera [16], and ranging in age from the Early Jurassic to the Early Cretaceous. Compared with other families of sawflies, Xyelotomidae are somewhat more disparate in morphology than other early symphytan lineages, as evidenced by their wide range of venational patterns and antennal shapes. Monophyly of the family has never been established and they are putatively early relatives of the superfamily Tenthredinoidea, and the apparent disparity may reflect nothing more than the fact that they are a grade at the stem of the tenthredinoids.

Gao et al. summarized the evolutionary patterns of changes observed in vein Sc across lower Hymenoptera based on several xyelotomid fossils which exhibit a diversity of venational forms, further argued that xyelotomids are merely an artificial paraphyletic assemblage [6]. Among early Xyelidae, such as *G. quadrifurcata*, the forewing Sc is four-branched, and this condition is reduced to three-branched in *Abrotoxyela*, *Xyelites* and *Shartexyela* [17, 18], and two-branched in other genera [12, 18–20]. In Xyelotomidae, vein Sc varies widely from the groundplan of a free Sc with two branches ending both on C and R (in *Pseudoxyela*) to entirely lost (in *Leridotoma*), with various intermediate states such as a complete vein with either anterior or posterior branches lost entirely or partially (*Paradoxotoma*), or else split into a basal longitudinal part (normally ending on R, or sometimes lost totally), and a distal crossvein-like part (for more details, see [6]). In its ultimate form, Sc is wholly unrecognizable in *Leridatoma* [21] and some extant Tenthredinoidea, the result of complete fusion with R as evidenced in developmental studies of living taxa [22, 23].

Xyelids are documented as the sister-group of all extant hymenoptera [8, 24], and have very interesting antennal structure. The first antennal flagellomere is elongate and thickened, with the remaining flagellomeres of a form similar to the flagella of many living hymenopterans and variable in total number of articles, but often with more than 15 flagellomeres. Although retaining a xyelid-like antennae, xyelotomids have a more apicomorphically reduced number of flagellomeres (less than 8), suggesting a hypothetical evolutionary pathway from xyelid-like antennae to common filiform antennae [6, 25].

### Asymmetrical wings – venation or size and shape

The holotype specimen of *Aethotoma aninomorpha* gen et sp. nov. here exhibits its own, unique asymmetrical venation, that is, the crossvein 2r-rs of the right forewing forms a loop, then forks into 2 long braches reaching Rs (Fig. 3c and f), while the 2r-rs of the left forewing forks into 2 short braches reaching Rs (Fig. 3b and e), in contrast to a linear 2r-rs present in other fossil and extant sawflies [26]. The previously described xyelotomid, the holotype specimen of *Xyelocerus diaphanus* Gao, Ren & Shih, 2009, from the same locality, possesses an small cell of vein Rs between veins R and M in the left forewing (Fig. 3h, i and k), but not in the right forewing (Fig. 3h, j and l) [6].

**Fig. 4:**
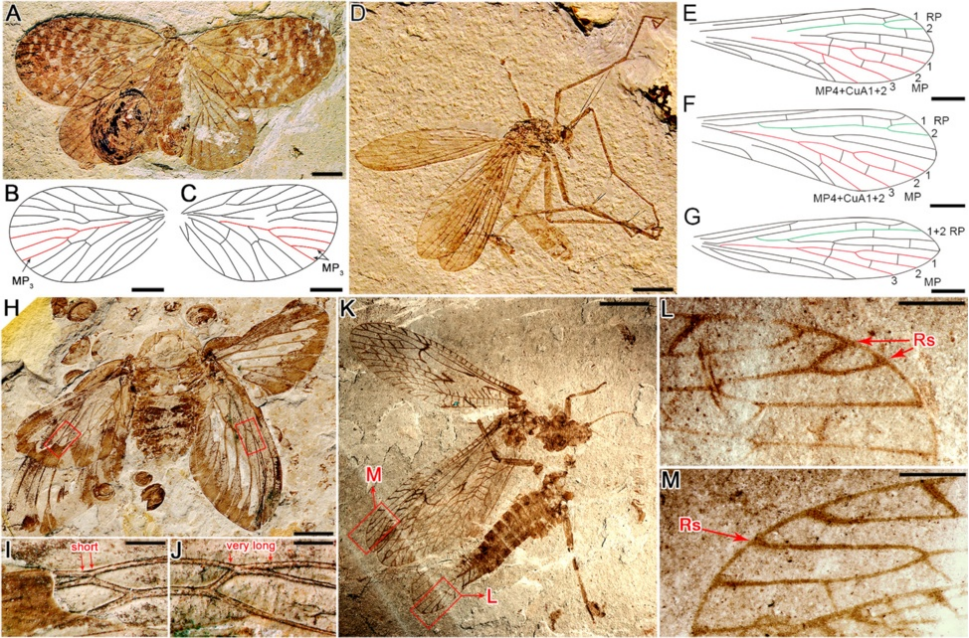
Several examples of the fossil insects with the wings possessing venational asymmetry reported from the Mesozoic of China. **a**–**c**, *Paristopsyche angelineae* within Mecoptera showing the different branches of vein MP3 between left and right forewings. **a**, photograph of the specimen; **b**, line drawing of left forewing, vein MP3 with typical one branch; **c**, line drawing of right forewing, vein MP3 with two branches. **d**–**g**, *Exilibittacus lii* within Mecoptera showing the different vein RP and MP on fore and hind wings. D, photograph of the specimen. **e** and **f**, right and left forewings with RP1+2 forking and MP with four branches. **g**, left hind wing with RP1+2 not forking and MP with three branches. **h**–**j**, *Synapocossus sciacchitanoae* within Hemiptera showing the RP coalesced with M1 on right and left forewings. **h**, photograph of the specimen. **i**, left wing with the Rs coalesced with M1 at a point. **j**, right wing with the RP coalesced with M1 for about 1 mm. **k**–**m**, *Sinosharaperla zhaoi* within Plecoptera showing different Rs forking on right and left forewings. **k**, photograph of the specimen. **l**, part of right hind wing showing Rs with two terminal branches. **m**, part of left hind wing, Rs with only one branch. Scale bar represents 5 mm (**h** and **k**), 2 mm (**a**–**d**), 1 mm (**e**–**g**, **i**, **j**, **l** and **m**)

A few other cases of clearly recognizable venational asymmetry on individual insects have been documented from the Mesozoic of China. *Paristopsyche angelineae* Qiao, Shih, Petrulevičius & Ren, 2013, a choristopsychid in Mecoptera, has vein MP_3_ with two branches on the right forewing, instead of the typical one branch on the left forewing [27] (Fig. 4a–c); the bittacid *Exilibittacus lii* Yang, Ren & Shih, 2012 has RP+MA and MP of its left hind wing bearing only three branches and RP1+2 and MP3 not forking, even though RP+MA and MP of its left and right forewings with the typical four branches of most hangingflies [28] (Fig. 4d–g); in the paratype (CNU-HEM-NN2007008p/c) of *Synapocossus sciacchitanoae* Wang, Shih & Ren, 2012, a palaeontinid in Hemiptera, from Daohugou, China, the right forewing has 1 mm of coalescence of RP with branch M1, but only a point contact on the left forewing [29] (Fig. 4h–j); and differences between the left and right hind wings of the same individual have been described in the plecopteran *Sinosharaperla zhaoi* Liu, Sinitshenkova & Ren, 2007 [30] (Fig. 4k–m).

Asymmetrical shape and size of the left and right wings on a specimen of *Epicharmesopsyche pentavenulosus* Shih, Qiao, Labandeira & Ren, 2013 seems to be a common condition for mesopsychid taxa (Mecoptera) from northeastern China [31]. Notably, the asymmetrical wing shape and size of the left wings are broader than the right wings, which seems to be common for mesopsychids as reported by Ren et al. [32, 33] and in other specimens collected after the publication of these two papers [31]. Asymmetrically unequal wings also occur in the dipteran family Ptychopteridae, as reported for *Eoptychopterina elenae* Ren & Krzemiński, 2002 [34], and for *E. postica* Liu, Shih & Ren, 2012 [35].

## Conclusions

Given the vast diversity of species, typically high number of individuals, and global distribution, insects may serve as an excellent group from which to observe fluctuating asymmetry in the fossil record. However, the exploration for and study of asymmetry in the fossil record of insects have been minimal [36, 37], typically because there is insufficient material from which to work, as clear asymmetry occurs at a much lower rate than unaltered individuals [38]. Some of the best studies of asymmetry in fossil insects have examined the irregular variation of wing veins in fossil termites [39, 40] and cockroaches [41], which all focus on the fossil insects with complex and primary venational pattern. The new finding of *Aethotoma aninomorpha* gen. et sp. nov. and previously documented asymmetrical examples from the Mesozoic of the Northeastern China provide a glance at early occurrences of venational variability and instability, or possibly aberrational development, for insects in the late Middle Jurassic. It is suggested that more attentions should be paid to recognize and distinguish whether a venational character is an aberration or a diagnostic character in fossil insect classification, when we carry out taxonomy research based on a single wing of the afore-mentioned groups of insects [42].
